# THE RELATIONSHIP BETWEEN MEDIA PORTRAYALS OF OLDER PEOPLE AND PERCEPTIONS OF INTERGENERATIONAL CONFLICT

**DOI:** 10.1093/geroni/igad104.2711

**Published:** 2023-12-21

**Authors:** Soondool Chung, Ahyoung Lee, Ju-Hyun Kim

**Affiliations:** Ewha Womans University, Seoul, Republic of Korea; Ewha Womans University, Seoul, Republic of Korea; Chungnam National University, Daejeon, Republic of Korea

## Abstract

As South Korea experienced rapid social change in several decades, there are lots of gaps in historical and cultural experiences of younger people and older people. In addition, with rapid population aging, generational tension became stronger due to limited resources and opportunities. This study examined the relationship between media portrayals of older people and intergenerational conflict and the mediating role of prejudice against older people in the relationship in South Korea. Also, the relationship between trust and intergenerational conflict was examined. Finally, the buffering effect of trust in the relationship between media portrayals of older adults and intergenerational conflict was examined. Secondary data analyses were conducted using Age Integration and Intergenerational Solidarity survey data of Korean adults aged 20 and over with a total of 1,000 cases. The findings revealed that prejudice against older people mediated the relationship between the media’s portrayal of older people and perceptions of intergenerational conflict. Trust showed a significant negative relationship with intergenerational conflict. Finally, trust had a buffering effect on the media’s negative portrayal of older adults and intergenerational conflict. For people with lower levels of trust, there was a significant positive relationship between negative media portrayals of older adults and intergenerational conflict. Further implications for practice to reduce intergenerational conflict were discussed.

